# Separation of Sensitized and Non-Sensitized RBCs: Sephadex-Based Cell-Affinity Adsorbents

**DOI:** 10.1371/journal.pone.0045583

**Published:** 2012-09-21

**Authors:** Jingchun Liu, Yan Wang, Fuping Liu

**Affiliations:** 1 The Blood Group Reference Laboratory, Dongguan Blood Center, Dongguan, Guangdong, P. R. China; 2 Department of Urology, Yale University School of Medicine, New Haven, Connecticut, United States of America; 3 Department of Pediatrics, Dongguan People's Hospital, Dongguan, Guangdong, P. R. China; University of Colorado, United States of America

## Abstract

**Introduction:**

In transfusion medicine, antibodies that cause RBCs positive DATs, may interfere with patients' phenotyping. Traditionally, these antibodies were removed using various antibody elution methodologies. However, the elution agents and conditions used have been only partially successful; and no one method is superior. The purpose of this study was to develop a general and efficient method to separate non-sensitized from sensitized RBCs using Sephadex-based cell-affinity adsorbents.

**Methods:**

First, we coupled Sephadex support with Staphylococcal Protein G (SpG) with or without NHS. Then we simulated clinical conditions by mixing differe∏nt ratios of sensitized and non-sensitized RBCs in vitro. Sensitized cells were prepared by mixing antibody with corresponding antigen-positive RBCs. Finally, we checked the sensitization status of absorbed RBCs after absorption with modified Sephadex support.

**Results:**

The number of sensitized RBCs bound to Sephadex-based cell-affinity adsorbents is approximately 5×10^8^ RBCs/mL support. Activated Sephadex could separate sensitized from non-sensitized RBCs. **Conclusion** Sephadex-based cell-affinity adsorbents with an NHS spacer arm have bigger capacity for binding RBCs than unmodified Sephadex. The Sephadex-based cell-affinity adsorbents readily separate non-sensitized RBCs from sensitized RBCs, thus providing a new strategy to type the blood for transfused patients.

## Introduction

In transfusion medicine, there are many reasons why patients present with a positive direct antiglobulin test (DAT). These include haemolytic transfusion reactions, autoimmune hemolytic anemia (AIHA), and hemolytic disease of the newborn (HDN). In these conditions, determination an accurate RBC phenotype can be problematic because, if RBCs are already coated in vivo with immunoglobulin, complement, or both; all tests performed will be positive using the indirect antiglobulin test (IAT). Alloimmunization is a common phenomenon after transfusion, with an estimated incidence of 0.5%, increasing to 20–60% in chronically transfused patients [1]. As alloantibodies can cause hemolysis of transfused RBCs, their specificities must be identified for further compatible transfusions. Phenotyping by hemagglutination assay less than three months after transfusion can be difficult and often impossible because the antibodies that can cause RBCs positive DATs and produce mixed-field agglutination may interfere with patients' phenotyping. There are very few IgM directly agglutinating reagents available for the clinically significant antibodies (i.e. anti-K, -Jk^a^, -Jk^b^, -S, and -Fy^a^) [2]. In order to type the blood group correctly, the traditional method is to remove antibody but leave intact red cells. There are some procedures available. A mixture of dithiothreitol and cysteine-activated papain, completely denatures Kell, Duffy, and MNS system antigens [3]. Microwave irradiation is difficult to regulate and may physically alter RBCs [4], [5]. The citric acid elution method is commonly used, but a major drawback is that antigens of the Kell system are significantly weakened by this method [6], [7]. Other method, such as CPD (CPD is an anticoagulant-preservative approved by the FDA for 21-day storage of RBCs) and enzyme/reducing agent treatments can cause damage to the RBCs, resulting in the loss of some RBC antigens and possible invalid typing results. Additionally, CPD may not totally remove the coating autoantibody from the RBCs and it does not remove complement component 3 (C3) [6]. RBCs treated with the reagent combining both these chemicals therefore have limited applications for use in phenotyping studies. Each elution reagent and condition has been somewhat successful; however, no one method is superior [2], [8]–[10].

At present, the most widely employed techniques for isolation of cell populations are affinity-based separations that make use of monoclonal antibodies or other specific ligands. Affinity cell separation techniques are used to quickly and efficiently isolate specific cell types from heterogeneous cellular suspensions, based on ligand-specific binding involving cell surface molecules [2], [7], [11], [12].

The purpose of this study is to develop a general and efficient method to separate non-sensitized from sensitized RBCs with Sephadex-based cell-affinity adsorbents.

## Materials and Methods

### Materials

Sephadex G-50 was purchased from Amersham Biosciences (Uppsala, Sweden). Staphylococcal Protein G (SpG), dimethylpimelimidate (dihydrochloride), *N*-Ethyl-*N*′-(3-dimethylaminopropyl) carbodiimide (EDC), CDI (Carbonyldiimidazole) were obtained from Sigma (St. Louis, MO, U.S.A.). Rabbit anti-(human RBC) antiserum was obtained from the SHPBC (Shanghai Hemo-Pharmaceutical & Biological Co., Ltd, Shanghai, China). N-hydroxysuccinimide (NHS) ester was obtained from Fluka AG (Buchs, Switzerland). Anti-D, -E, -Jka, -Fya, -K are obtained from Lorne laboratories limited, UK. Coomassie Brilliant blue dye was purchased from Bio-Rad Company (Hercules, CA. USA). The autoantibodies anti-D, -c and phenotyped RBC were identified in our laboratory.

### Methods

#### Preparation of NHS-activated glycine-Sephadex G-50

Sephadex G-50 was carboxylated by derivatization with glycine, and the resulting glycine-sephadex G-50 was activated with EDC and NHS as described by Besselink [13].

#### Preparation of SpG-NHS-glycine-Sephadex [14]

Dry NHS-activated glycine-Sephadex G-50 was suspended in 0.1 M NaCO3, pH 8.5 at a dry substance content of 31% (w/v). Immediately after suspending the support, SpG was added at 1 mg/mL. The suspension was mixed for 2–4 h at room temperature (RT) using an overhead mixer. The mixture was washed three times in 200 mL of 0.1 M NaCO3, pH 8.5, by sedimentation, and suspension of the support in 50 mL of 0.1 M sodium phosphate, pH 7.4, containing 1 M ethanolamine to block free reactive side chains.

#### SpG attachment directly by water-soluble CDI coupling

The swelled support was washed with 0.5 M NaCl and 0.1 M sodium phosphate, pH 4.5. And one mL of 1 mg/mL SpG in 0.1 M pH 4.5 sodium phosphate was added. Then 1 mL of the CDI was slowly added with gentle agitation. The mixture was incubated overnight on an overhead mixer at RT and then was washed with 500 mL of 0.1 M sodium phosphate, pH 7.2 using a glass filter.

#### Bradford protein assay [15]

The concentration of protein remaining in the supernatant of the support suspensions after protein coupling was determined with the Coomassie Plus Protein Assay Reagent. This assay is based on the Bradford method, which utilized the shift in absorbance of the Coomassie Brilliant Blue dye at 595 nm after binding of the dye to protein.

#### Preparation of sensitized and non-sensitized RBCs

Five mL O type homozygous D(+) RBCs concentrate was collected and washed three times with physiological saline (PS). One mL IgG anti-D was added and incubated for 30 min at RT. The RBCS were washed three times with PS and resuspended with SAG-M (150 mM NaCl/1.25 mM adenine/47 mM glucose/29 mM mannitol). The haematocrit was adjusted to approximately 30% by addition of SAG-M. The resulting suspension had a red-cell count of approximately 3.2×10^12^cells/L. For the other antibodies, the procedure is the same as that of anti-D and the RBCs used were homozygous and positive for the antibody tested.

The non-sensitized RBCs were prepared by incubating irregular-antibody free AB type serum for 30 mins and then washing three times.

#### RBCs-binding experiment

One g SpG-NHS-glycine-Sephadex was resuspended in 5 mL PBS and allowed to stand overnight. Both sensitized and non-sensitized RBCs in varying proportions were mixed with 0.2 mL of affinity support and the resulting suspension was shaken gently for 15 min. The support beads were allowed to sediment, the supernatant was aspirated and the sedimented support was resuspended after addition of PBS (5 mL). Washing was repeated four more times. Finally, the supernatant was aspirated as completely as possible and support-bound red cells were lysed by addition of 2.0 mL of deionized water. Hb (Hemoglobin) levels were measured by HiCN technique. The corresponding red cell number was calculated.

#### Separation of sensitized and non-sensitized RBCs

By mixing different ratios of sensitized and non-sensitized RBCs, we created an in vitro model to replicate clinical transfused patients' samples. The sensitized RBCs were prepared as described above.

Combinations of antigen-positive cells and antigen-negative cells were generated in 75 to 25 percent, 50 to 50 percent, and 25 to 75 percent proportions. The antibodies tested included anti-D, -E, -Jka, -Jkb, -K -Fya and –Fyb (Table 1). Cord blood and outdated blood served as negative and positive reticulocyte controls. The concentration of RBC suspension mixtures were adjusted to about 0.8% by PS. A 0.5 mL aliquot of RBC suspension was added to 1 mL activated Sephadex and centrifuged 10 min at 50 g. The support and RBCs were suspended and the beads allowed to sediment. The supernatant contained the non-sensitized RBCs. The RBCs in supernatant were subject to a DAT test to identify the status of sensitization. The result was considered successful if the cells remaining after absorption were non-sensitized and unsuccessful if the sensitized cells were not completely absorbed.

**Table 1:** Results of separation mixed sensitized and non-sensitized RBC by Sephadex-based cell-affinity adsorbents.

Sample ID	antibody specificity	result			
		10%	25%	50%	75%
1	Alloanti-D	√	√	√	√
2	Alloanti-E	√	√	√	√
3	Autoanti-D	√	√	×	×
4	Alloanti-Jka	√	√	√	√
5	Alloanti-Fya	√	√	√	√
6	Monoclone anti-K	√	√	√	×
7	Alloanti-c	√	√	√	√

“√” means successful, “×” means unsuccessful.

#### Microscopy observation

Affinity support, incubated with sensitized RBCs, was fixed with 4% formaldehyde/2% glutaraldehyde in PBS for 2 days at 4°C. After fixation, the samples were examined with Carl Zeiss Axioskop 40 upright microscopy (Carl Zeiss MicroImaging Inc, Germany). The images were analyzed by Auto-Montage pro software (Synoptics Ltd, Cambridge, England) to produce one focused montage image.

## Results

### Immobilization of SpG

The amount of support-immobilized SpG increased as more protein was added (Table 2). The maximal concentration of immobilized protein, as deduced from the depletion of the supernatant SpG, was nearly 275 µg SpG/mL support. The derived SpG-glycine-Sephadex immobilized SpG was nearly 220 µg SpG/mL support.

**Table 2:** Amounts of SpG immobilized to glycine-Sephadex G-50 (NHS-activated or not).

SpG added (mg/mL of support)	Sephadex-Gly (µg SpG/mL of support)	Sephadex-Gly-NHS(µg SpG/mL of support)
0	0	0
0.05	44	46
0.1	93	95
0.15	125	138
0.2	151	182
0.25	182	230
0.3	211	272
0.5	221	275

### Sensitized cell binding

The number of sensitized RBCs bound to SpG-NHS- Gly-Sephadex support increased as more sensitized RBCs were added (Table 3). The affinity support could bind up to approximately 5×10^8^ RBCs/mL of support. The SpG coated unmodified support bound only sensitized 2.5×10^6^ RBCs/mL support.

**Table 3:** Amounts of RBCs bound to derived support.

Sensitized RBCs suspension*	Sephadex-Gly-NHS-SpG(×10^7^RBC/mL of support)	Sephadex-Gly-SpG(×10^6^RBC/mL of support)
10 µL	3.10	2.51
20 µL	5.81	2.48
40 µL	12.6	2.36
100 µL	29.1	2.52
200 µL	48.3	2.51
400 µL	50.2	2.53
600 µL	50.1	2.50

* Concentrations of RBC about 3.2×10^12^cells/L.

**Figure 1:**
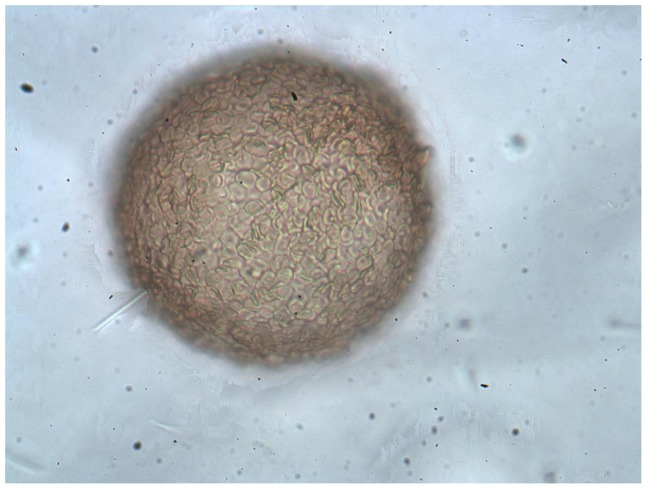
Micrograph of sensitized RBCs bound to Sephadex (×400).

Micrographs illustrated that after incubation of the affinity support with sensitized RBCs, the affinity beads were coated with cells (Figure 1). Agitated RBCs bound to the derived support without NHS were more easily eluted from the Sephadex surface than the agitated RBCs with NHS (data not shown).

### Separation of sensitized and non-sensitized RBCs

**Figure 2:**
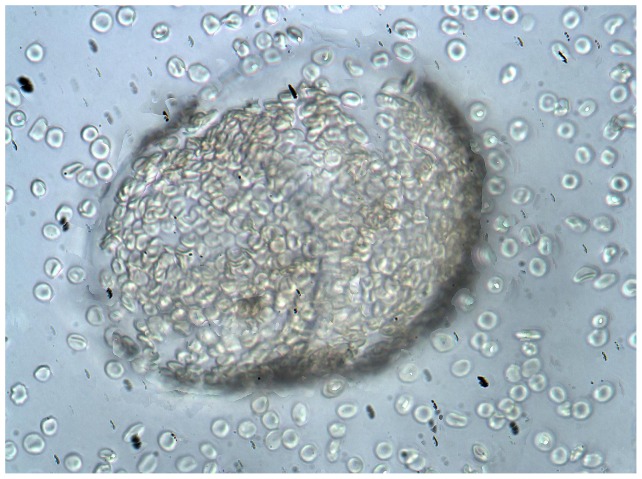
Separation of RBCs from a patient. The sensitized RBCs bound to the affinity support, while the non-sensitized RBCs remained in the supernatant (×400).

After being absorbed by Sephadex-based cell-affinity adsorbents, mixtures of sensitized and non-sensitized RBCs could readily be separated from each other. The non-sensitized cells were unbound and sensitized RBCs were bound to the derived Sephadex support (Figure 2). There was clear differentiation of antigen-positive and antigen-negative cells when the antigen-positive population was the major or minor population. Similar results were obtained with equal mixtures of antigen-positive and -negative cells for antigens D, E, Jka, Jkb, K Fya and Fyb using alloantibodies. When we used autoantibodies and monoclonal antibodies (table 1), we could not separate the antigen-negative RBC from the antigen-positive RBCs when they were 75 percent of the population. The micrographs show bound RBCs and free RBCs (Figure 2). If those free RBCs DATs are negative, then the RBCs are non-sensitized. It means the sensitized RBCs were absorbed by support.

## Discussion

Solid phase derivatized with cell-specific antibodies has been used in preparative techniques such as immunoaffinity for the isolation or depletion of specific types of cells from heterogeneous cell suspensions. Staphylococcus Protein G (SpG)-derivatized matrices provide immobilized ligands that can bind antigen more efficiently compared with antibodies that are directly coupled to the solid phase [16]. We selected Sephadex G-50 as matrix because its exclusion limit is 15 kDa. SpG is 42 kDa and it will be restricted to the outer surface of the matrix. This is the reason the antibody binding capacity to Sepharose CL 4B is remarkably higher than the Sephadex [13], [16]. In this study, the separation target is RBC that is too big to enter the beads. Using Sephadex necessitates the use of relatively less SpG.

Many different procedures have been described to activate hydroxylic groups on polymers, like Sepharose, dextran, and cellulose. One well-known method is carbodiimide activation of support-bound carboxylates, in the presence of NHS [17]. It placed the ligand at a distance from the matrix backbone and reduced the sterical hindrance of the ligand. In this study, the NHS modified support binds 100 times the number of RBCs while SpG binding is similar between NHS modified and unmodified support. It demonstrated that the NHS spacer had little effect on coupling SpG but greatly improved the binding of sensitized RBCs to derivatized Sephadex. Steric hindrance often occurs between the ligand and the target to be isolated, causing reduced reactivity or non-reactivity between target and the affinity ligand. Coupling the ligand to the support with a spacer arm like NHS could reduce the steric hindrance greatly. The RBCs are negatively charged, which results in difficulty in their binding to the derived support. Because NHS extends the distance between the ligand and the support, it is easier for sensitized RBCs to react with SpG and the strength of binding is stronger when the support is not modified with NHS.

Patients who receive transfusion develop red cell antibodies that may require phenotypically-matched allogeneic blood for subsequent transfusions to prevent further immunologic stimulation [6], [18]. Phenotyping of recently transfused patients requires separation of the recipient and donor cells and it is a serologic challenge. Samples from these patients have a mixture of their own and donors blood cells. Current methods include isolating the reticulocytes from the patients' cells by centrifugation, flow cytometry or immunomagnetic beads [19]–[21] or using conventional adsorption procedures to remove autoantibodies from intact RBCs stated as previously stated. Sephadex-based cell-affinity adsorbents is a promising method to separate non-sensitized RBCs from sensitized RBCs. It doesn't involve harsh physical or chemical methods or destroy the RBCs antigens. Therefore, it presents an alternative to separate the sensitized cells in patients. In this study, we used Sephadex-based cell-affinity adsorbents to separate the non-sensitized cells successfully for an alloantibody model. The method was adaptable to an array of red cell antigens using alloantibodies, thereby maximizing the probability of identifying a donor-recipient mismatch that could be exploited as a marker. Samples that contained the exceptions, i.e. the autoantibodies or monoclonal antibodies, produced inconsistent and unreliable results. One reason for this inconsistency may be because the autoantibodies non-specifically bind to antigens or SpG. In patients, the autoantibodies in the serum bind to the SpG and the sensitized RBCs were excluded; another reason is, in the AIHA patients, almost all of the cells were coated with antibodies and there were very few non-sensitized RBCs in the serum. This method is useful when there are mixed populations of antibody coated and uncoated red cells, such as in a hemolytic transfusion reaction, or useful as an indirect antiglobulin technique for separation of mixed cell populations where there is not coating alloantibodies, as in the multiply transfused patients. Some monoclonal antibody ABO grouping reagents are sensitive to changes in pH and osmolality [6], there are no such studies for other blood grouping reagents. In my opinion, maybe some of them have the same characteristic. In the adsorption, the pH and osmolarity are inconstant; some of the antibodies were detached from RBC. That is the reason for low separation efficiency for monoclonal anti-K.

The affinity separation technique has high specificity for the target cells, with high yields in short periods of time coupled with a simple procedure [11], [22]. In transfusion medicine, typing the patients' blood group correctly is very important. Using physical methods like heat or chemical methods to remove the Antibodies from the RBCs has some disadvantages. Using Sephadex-based cell-affinity adsorbents to separate sensitized and non-sensitized RBCs can overcome the problems. To ensure the correct identification of blood group and the safety of transfusion, the method in this study could be a new choice. At present, commercial affinity matrix and affinity column can be obtained from suppliers making this method more available and convenient.
